# From sexual harassment to sexual assault: Prevalence and correlates of sexual trauma in the French military

**DOI:** 10.1371/journal.pone.0259182

**Published:** 2021-11-17

**Authors:** Caroline Moreau, Dina Bedretdinova, Sandrine Duron, Aline Bohet, Henri Panjo, Nathalie Bajos, Jean Baptiste Meynard

**Affiliations:** 1 Soins et Santé Primaire, CESP Centre for Research in Epidemiology and Population Health, U1018, Inserm, Villejuif, France; 2 Department of Population, Family and Reproductive Health, Johns Hopkins Bloomberg School of Public Health, Baltimore, MD, United States of America; 3 French Military Center for Epidemiology and Public Health, Marseille, France; 4 INSERM, UMR S 912, « Economic and Social Sciences for Health and Processing of Medical Information » (SESSTIM), Marseille, France; 5 IRIS Institute of Interdisciplinary Research on Social Issues, U997 Inserm—EHESS, Aubervilliers, France; 6 French Military Medical Academy, Ecole du Val-de-Grâce, Paris, France; University of Bristol, UNITED KINGDOM

## Abstract

**Background:**

Sexual harassment (SH) is prevalent in military settings and dependent on the workplace environment. Few studies have investigated this issue in non-US military settings nor have examined how contextual and individual factors related to Military Sexual Trauma (MST) vary by gender.

**Methods:**

This study draws on a national sexual survey in the French military including 1268 servicemen and 232 servicewomen. We examined four sexual stressors (repeated sexual comments, sexual coercion, repeated unwanted verbal sexual attention and sexual assault (SA)) and two combined measures of verbal SH (comments, unwanted attention) and MST (all forms). We conducted multivariate logistic regressions to identify contextual and individual factors related to these outcomes.

**Results:**

36.7% of women and 17.5% of men experienced MST in the last year and 12.6% and 3.5% reported SA. Factors associated with verbal SH differed from those related to SA. The odds of verbal SH were elevated among men who had sex with men (OR = 3.5) and among women officers (OR = 4.6) while the odds of SA were elevated among men less than 25 years (OR = 3.5) and women with less than a high school diploma (OR = 10.9). The odds of SH increased by 20% to 80% when men worked in units with higher female representation, higher prevalence of MST (sexual comments, or sexual assault, coercion, repeated unwanted attention) and lower acceptance of women in the miliatry. The odds of SA also increased by 70% among men working in units with higher female representation and higher prevalence of sexual oppression. The odds of SA against women were particular high (OR = 5.7) in units with a high prevalence of sexual assault, coercion, or repeated unwanted attention.

**Conclusion:**

MST is common in the French military, with women experiencing more severe forms than men. Our resuls call for programmatic action to reduce workplace factors related to verbal SH and SA in the French military.

## Introduction

Sexual harassment (SH) is a common experience in the workplace, although estimates vary widely, ranging from 40% to 75% of women and from 13% to 31% among men [1]. Earlier research has defined the contours and estimated the incidence of SH [2, 3], while a second generation of studies has focused on the circumstances and sequela of SH [4–6]. At the intersection between legal and psychological constructs [7, 8], the definition of SH is still a matter of debate, despite conceptual and psychometric advances in the 1990s. Fitzgerald’s Sexual Experience Questionnaire distinguishes three dimensions: gender harassment, defined as “behaviors that convey hostile, offensive, and misogynist attitudes” [3], unwanted sexual attention defined as “incidents such as sexual imposition, touching, or repeated requests for dates” [9], and sexual coercion [3, 10, 11]. These domains have been refined overtime [12], but they still capture a continuum of severity and distinguish between legal entities of quid pro quo (sexual coercion) and hostile environment (gender harassment and unwanted sexual attention).

Research on the causes of SH indicates that it is prevalent across ages, socioeconomic groups, and cultures [13], suggestive of a “universal” phenomenon [14]. Fitzgerald and Drasgow’s conceptual model theorizing the causes and consequences of SH draws attention to the situational characteristics increasing SH, including the workplace climate and gender structure [3, 15]. Supporting this framework, a meta-analysis of 41 studies indicates a strong effect of organizational climate and job gender context on SH victimization [4]. Tolerance for SH and lack of sanctions are predisposing factors [16], while imbalanced sex ratio [4] and hegemonic masculinity norms valuing toughness and aggression as the “epitome of masculinity” [17, 18] work in tandem to increase SH [19]. These contextual elements are generally considered as individual perceptions rather than group-level measures, resulting in over-estimation of associations [15]. A recent study applied group level measures to show that sexist climate in US military units increased individual risks of SH [20]. The study however, did not consider gender differences in these contextual effects. Few studies in fact, consider a Person-X-Situation model interacting the social context with individual characteristics. Pryor et al. applied such a model showing that the environmental climate had different effects for males and females in the US military [21, 22].

The prominence of SH in the military has received much attention in the US [23, 24] in the wake of the feminization of the profession and higher SH prevalence compared to civilian populations [2, 6]. In the military context, SH is often assessed as a component of military sexual trauma (MST), which represents a continuum of harm from sexism to sexual assault (SA) [24, 25].The 2018 US estimates among active duty military personnel indicated that 24.2% of servicewomen and 6.3% of servicemen had experienced SH (excluding assault) in the past 12 months while 6.2% of women and 0.7% of men reported SA [26]. Cultural norms valuing hegemonic masculinity likely contribute to higher SH in military populations [19], while maculinized environments that tolerate SH behavior and lower sociocultural power contribute to heightened risk of SH within military populations [27]. Few studies have explored SH in other military settings. One Swedish study indicated that 1.9% of female cadets reported sexual quid-pro-quo [28] and 83% reported gender harassment and unwanted sexual attention in a 24 months span. A South Korean study reported a prevalence of SH of 5.7% among female military personnel [29].

The present study aims to extend our current knowledge of MST in military populations outside of the United States, by 1) evaluating the incidence of a spectrum of experiences from sexist remarks, unwanted verbal sexual attention, sexual coercion, to SA among women and men in the military and by 2) exploring individual and contextual factors that increase the risk of experiences these events according to gender.

## Methods

### Study design and participants

We draw on data from the COSEMIL study, conducted in 2014–2015 among a national probability sample of 1,500 active duty members (1,268 males and 232 females). The design of the COSEMIL study is described in more detail elsewhere [30]. Briefly, COSEMIL followed a two-stage probability sampling design, based on the selection of 18 military units and 120 active duty members within each unit. Military units were randomly selected from all units after stratification by branch (Army, Navy, Air force) and location (Mainland France, overseas). Within each unit, 120 service members aged 18 and over were randomly selected with over representation of women (1 woman for 5 men). A total of 1,971 servicemen and women were invited to attend an information session describing study goals and procedures and 1,692 attended (some had conflicting schedules). After the information session, participants were invited to provide written consent to participate. A total of 178 individuals refused participation (145 males and 33 females) and 14 questionnaires were lost due to software deficiency. The analytic sample includes 1500 participants (participation rate 76%). Individuals who were excluded were no different in terms of age, military rank, number of years in the military or deployment history but were more likely to be in the Navy. Post-stratification weights accounted for this non-response. The survey was approved by the Commission Nationale Informatique et Liberté (N° 2014–100).

### Measures

Participants self completed a 37-minute questionnaire on laptops; providing socio-demographic information, and information on their sexual attitudes and practices. The survey was pre-tested in a pilot study among 50 service members to assess the duration of the questionnaire and question comprehension. Building off of the DoD-SEQ instrument [31] and the French national survey on violence (Virage) [32], the survey included 7 items assessing receipt of sexual comments, sexual coercion unwanted verbal sexual attention and SA. Questions from the 2006 French sexual health survey [33] were used to explore lifetime SA including unwanted sexual contact, attempted and forced intercourse. A question on forced insertion of an object or finger in the vagina or anus was also added and included in the definition of sexual assault. Information about timing, perpetrator and context of each event, allowed identification of MST events that occurred in the military in the last 12 months. Twenty-nine of the 125 respondents who reported sexual assault did not provide information on timing and context of the event. Given high correlations between SA and other sexual stressors among respondents who reported SA in the military in the last 12 months, we reclassified 13 of the 29 individuals with missing information (11 women and 2 men) as having experienced SA in the military in the last 12 months if they reported other SH events in the military in the last 12 months. We also conducted sensitivity analysis by excluding these 29 cases. We constructed binary measures for each of the following sexual stressors occurring in the military in the last 12 months: repeated sexual comments, repeated unwanted verbal sexual attention, sexual coercion, and sexual assault. In line with the legal definition of SH, a single sexual comment or a single act of unwanted verbal sexual attention was not considered as SH. While some indicators could not be examined independently, given small sample sizes, we intended to identify factors related to different SH experiences from *verbal SH*, which captures non-physical forms of aggression [3] to *sexual assault*, involving physical agression. Thus, we created a composite indicator of *verbal SH* (repeated sexual comments, repeated unwanted verbal sexual attention). We also created an overall indicator of MST including all forms of sexual stressors. The different indicators represent different patterns of experiences along the continuum of harm, while MST provides an opportunity to compare our results with studies aggregating all forms of SH in a single indicator.

We explored individual and workplace environment factors related to these outcomes. Work environment factors included deployment in the last 12 months as well as group-level measures of sex composition, collegiality, gender attitudes, and sexual stressors measured at the unit level. Specifically, we averaged individual responses across members of a military unit (18 clusters) to assess the percentage who reported they formed a tightly knit group, the percentage who believed men had greater sexual needs than women and the percentage who indicated they opposed or had reservations about increasing female representation in the military. Group-level measures of repeated sexual comments and sexual oppression (representing crude and offensive behaviors that are sexual in nature [31] including unwanted sexual attention, coercion or sexual assualt) were also assessed by averaging responses of members of a participant’s unit excluding their own response.

### Analysis

We described the 12 months’ prevalence of each sexual stressor by frequency of occurrence. We identified individual factors related to each sexual stressor indicator as well as to verbal SH and MST using bivariate and multivariate logistic regressions. Next, we examined group-level variation in sexual stressors across the military units and performed multivariate logistic regressions (*ln (P /1-P) = a + b*_*n*_*X*_*n*_ *)* to identify individual and contextual factors related to verbal SH, sexual assault, and MST. We used cluster-robust standard errors to account for intra-military unit correlations. All analyses were stratified by sex and were weighted to account for unequal sampling probabilities and non-response.

## Results

The sociodemographic and sexual health characteristics of the study population are presented in Table 1.

**Table 1:** Characteristics of French active duty servicemen and women included in the COSEMIL survey (n = 1,500).

		Men		Women	
		n	%	n	%
Total		1268		232	
Age	18–24 years	239	19.1	55	17.8
	25–29 years	264	23.6	68	36.6
	30+ years	765	57.3	109	45.6
Current partner	Cohabitating all the time	768	58.0	124	58.8
	Cohabiting part of the time or Non-cohabitating	283	24.4	64	23.1
	No partner	216	17.6	43	18.1
Same sex partnership	No	1233	98.3	200	84.6
	Yes	17	1.7	32	15.35
	Never had sex	18	1.2		
Children	Yes	705	54.8	92	37.5
	No	561	45.2	140	62.5
Place of birth	Mainland France	1099	86.3	202	88.6
	Overseas France/Foreign country	169	13.7	30	11.4
Level of education	<High school	542	42.8	69	30.0
	High school graduation	468	34.5	114	46.1
	>High school	256	22.7	48	23.9
Financial situation	No problem	589	43.6	122	48.3
	Tight or Difficult	674	56.4	109	51.7
Military branch	Army	580	61.7	73	39.6
	Air force	381	18.3	97	43.6
	Navy	307	20.0	62	16.8
Military rank	Officer	119	12.1	14	6.3
	Non commissioned officer	587	44.6	99	46.8
	Enlisted personnel	562	43.3	119	46.9
Number of years in the military	<5 years	232	19.7	53	21.4
	> = 5 years	1032	80.3	179	78.6
Deployment in the last 12 months	No	892	65.5	193	87.3
	Yes	376	34.5	39	12.7

Altogether 14.6% of men and 30.3% of women reported repeated sexual comments that put them ill at ease in the workplace in the last 12 months (Table 2). Likewise, repeated unwanted verbal attention was more frequent among women (8.4%) than men (0.9%). Sexual coercion was less common, described by ten women (4.9%) and 4 men (0.4%). The gender gap was also evident in the case of SA, mostly in the form of unwanted sexual contact, reported by 12.6% women and 3.5% men. Four women (1.7%) and six men (0.5%) reported attempted or forced sex in the context of their work in the last 12 months. Altogether 36.7% of women and 17.5% of men experience MST, including any form of sexual stressor in the last 12 months.

**Table 2:** Measures and prevalence of *Sexual harassment*, *receipt of sexual comments*, *sexual coercion*, *unwanted sexual attention and assault* in the military work environment in the last 12 months by gender, n (%).

In the last 12 months in the context of your work	Men (n = 1268)			Women (n = 232)		
	No	Once	>Once	Never	Once	>Once
**Sexist or sexual comments**						
You heard sexist comments or jokes that made you uncomfortable?	1115 (86.8)	44 (3.4)	102 (9.8)	161(69.1)	19 (7.5)	52 (23.4)
You were put ill at ease by pictures of a sexual nature	1175 (91.6)	33 (3.8)	56 (4.6)	198 (86.2)	9 (3.8)	25 (1.0)
Someone has had sexual remarks or attitudes that have put you ill-at-ease–ex: questions about private life, salacious remarks, looks that undress, mimes of sexual gestures?	1163 (92.1)	36 (3.2)	62 (4.7)	167 (74.0)	20 (7.6)	45 (18.4)
***Any sexual comments (yes/no)***	**224 (20.1)**			**94 (40.8)**		
***Any repeated sexual comments (yes/no)***	**159 (14.6)**			**67 (30.3)**		
**Unwanted verbal sexual attention**	1128 (97.5)	24 (1.6)	13 (0.9)	197 (86.2)	14 (5.4)	19 (8.4)
Someone insistently made you sexual proposals, despite your refusal?						
***Any Unwanted verbal sexual attention (yes/no)***		**37 (2.5)**			**33 (13.8)**	
***Any repeated unwanted verbal sexual attention (yes/no)***		**13 (0.9)**			**19 (8.4)**	
***Verbal SH (yes/no***) (repeated sexual comments or repeated verbal unwanted attention)	**166 (15.1)**			**71 (32.0)**		
**Sexual coercion**						
You were suggested a reward or special treatment if you had sexual relations	1263 (99.9)	1 (0.1)	1 (0.0)	227(97.4)	4(2.4)	1(0.2)
You felt threatened if you were not sexually cooperative,	1264 (99.9)	0 (0.0)	1 (0.1)	227 (96.4)	2 (1.2)	3 (2.4)
You were treated badly because you refused sexual relations	1262 (99.8)	1 (0.0)	2 (0.2)	229 (98.2)	3 (1.8)	0 (0.0)
***Any sexual coercion (yes/no)***	**4 (0.4)**			**10 (4.9)**		
**Sexual assault**						
Someone touched your breasts, your buttocks, squeezed you, cornered you to kiss you, rubbed themselves against you against your will?	1223 (97.1)	21 (1.6)	22 (1.4)	214 (92.2)	10 (4.4)	8 (3.4)
Attempted or forced sex in the last 12 months including forced insertion of an object or finger in the vagina or anus	1262 (99.5)	6 (0.5)		228 (98.3)	4 (1.7)	
***Any Sexual Assault (yes/no)***	**49 (3.5)**			**28 (12.6)**		
(any touching, attempted or forced sex including with object or finger)						
***Any MST***	**200 (17.5)**			**80 (36.7)**		
(repeated sexual comments, repeated verbal unwanted attention, sexual coercion or sexual assault)						

**Fig 1:**
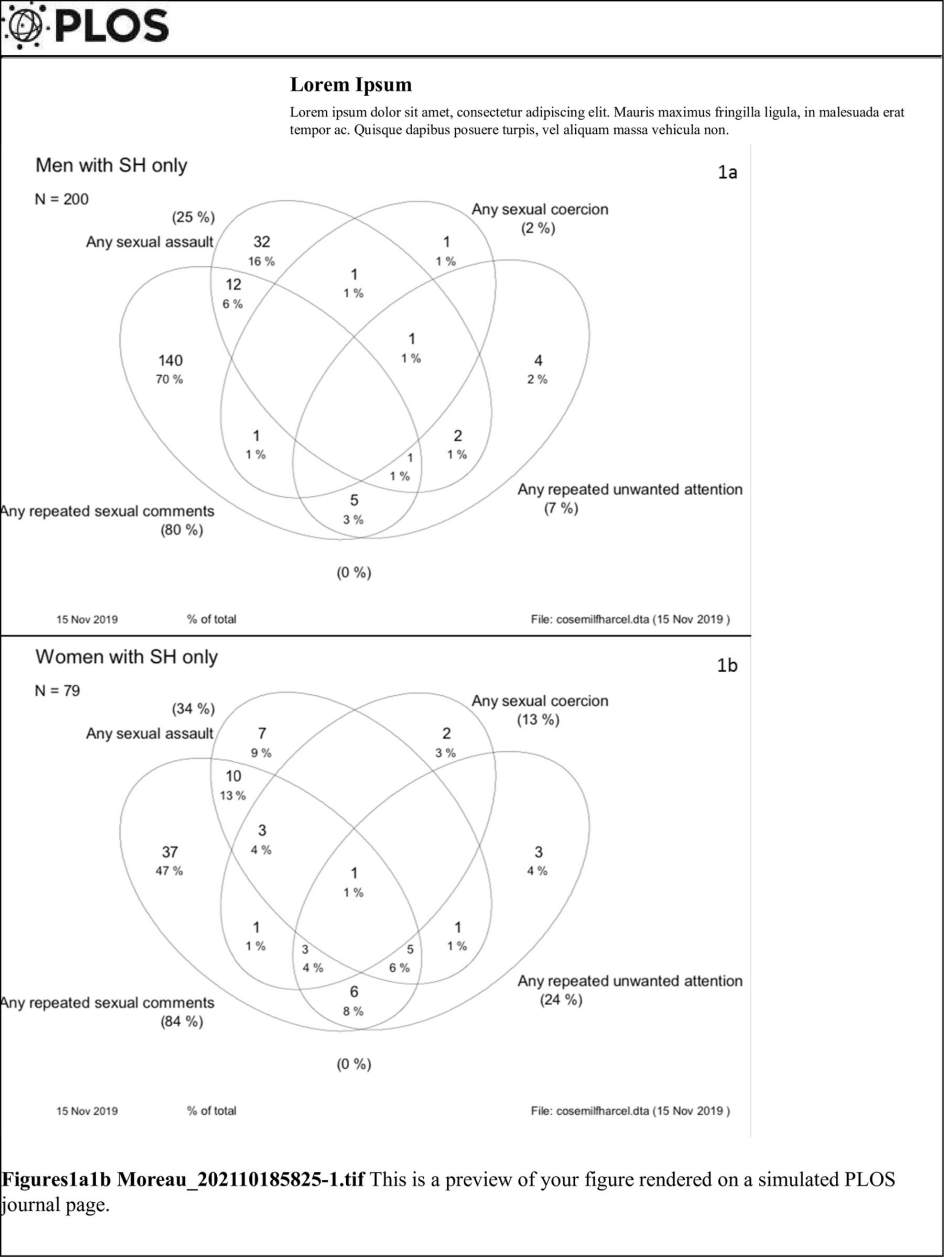
a, b: Distribution of type of sexual stressors experiences among service men and service women who report any MST event.

Thirty four percent of women and 16% of men who reported MST, experienced several forms of sexual stressors, mostly as a combination of repeated sexual comments and sexual assault (13% of women and 6% of men) or repeated comments and repeated unwanted verbal attention (8% of women and 3% of men) (Fig 1A and 1B).

Frequency of sexual comments varied from 4.7% to 21.9%, across units while SA varied from 1.9% to 12.1%. Factors associated with these experiences varied by type of stressor and sex (Table 3). Younger, non-cohabitating and childless men were more likely to have experienced SA while older men and men who had sex with men were more likely to report repeated sexist comments contributing to greater risk of verbal SH. Younger women, on the other hand, were more likely to indicate instances of unwanted verbal sexual attention and SA while non-cohabitating women were more likely to be exposed to repeated sexual comments. Men from socially disadvantaged backgrounds, including enlisted servicemen and participants born overseas or abroad, were more likely to report SA. Socio-economic differences were more complex among women as the most socially disadvantaged (lower education and enlisted personnel) were more likely to experience SA while the most socially advantaged (higher education and officers) were more likely to report sexual coercion.

**Table 3:** Percentage of service members who report a *repeated sexual comments*, repeated *unwanted verbal sexual attention*, *sexual coercion*, *or sexual assault* in the military work environment in the last 12 months by gender and sociodemographic characteristics.

		Men				Women					
Total		% repeat sex comments	% sexual assault	% Verbal SH^a^	% MST^b^	% repeated sexual comments	% repeated verbal unwanted attention^c^	% sexual coercion	% sexual assault	% Verbal SH	% MST^b^
Total		14.6	3.5	15.1	17.5	30.3	8.4	4.9	12.6	32.0	36.7
Age	<25years	11.0*	6.7***	10.9*	15.7	39.3	14.1*	8.1	20.8*	40.8	42.8
	25–29 years	**13.5**	**5.1**	**14.2**	19.0	24.4	**5.3**	1.2	**15.9**	26.6	31.6
	> = 30 years	**16.2**	**1.7**	**16.9**	17.5	31.6	**8.7**	6.7	**6.8**	32.9	38.3
Cohabitation with current partner	Everyday	14.3	2.1*	14.9	16.2	23.5*	2.9	3.5	9.8	2.9	32.8
	Not every day	17.2	**4.7**	17.2	20.3	**39.8**	21.8	8.6	15.0	21.8	42.2
	No current partner	12.0	**6.3**	12.9	17.9	**39.6**	9.3	5.1	18.8	9.3	41.5
Children	No	12.8	4.9**	13.5	17.2	31.4	11.0	7.2	15.3	33.1	36.6
	Yes	16.0	**2.3**	16.4	17.8	28.6	4.1	1.2	8.2	30.2	36.8
Same sex partnership	No	14.3**	3.4	14.8*	17.3*	31.0	7.1*	3.7***	12.8	31.7	35.8
	Yes	**38.8**	9.3	**38.8**	**38.8**	26.8	**15.7**	**11.6**	11.6	33.8	41.2
	Never had sex	**5.4**	0.0	**5.4**	5.4						
Place of birth	Overseas/Foreign	16.1	5.1*	16.4	19.4	32.5	7.7	2.6	14.9	32.5	32.7
	Mainland France	14.3	**3.2**	14.9	17.2	30.0	8.5	5.2	12.3	32.0	37.2
Level of completed education	< High school	11.5	4.4	11.5	14.0	35.4	10.7**	6.3	23.9*	39.2	49.5*
	High school	14.7	3.5	15.6	18.5	30.9	**5.8**	1.4	**10.9**	32.0	**34.5**
	>High school	20.5	1.7	21.2	22.8	23.5	**10.7**	8.2	**2.1**	23.5	**23.5**
Financial situation	No problem	15.3	2.8	15.5	18.0	34.6	8.0	4.3	11.3	36.2	39.2
	Difficult	14.1	4.0	14.9	17.3	26.6	8.9	4.8	14.0	28.3	33.8
Army rank	Enlisted personnel	12.0**	4.8*	12.4*	16.2	30.7	10.5*	5.9***	22.1*	33.8	41.8*
	Non commissioned officer	**14.7**	**2.4**	**15.5**	16.5	27.2	**5.6**	**2.8**	**4.8**	27.7	29.6
	Officer	**23.3**	**2.7**	**23.3**	26.1	50.5	**14.2**	**14.2**	**0.0**	50.5	50.5
Army contract	permanent	17.5*	2.0	18.0*	19.7	34.0	1.1*	0.0^#^	**0.3** ***	34.0	34.0
	temporary	**12.8**	4.4	**13.3**	16.2	29.3	**10.5**	6.3	**16.1**	31.4	37.4
Number of years in the military	< = 5 years	11.4	5.8	11.3	16.3	40.6	13.7	8.8**	24.7*	41.8	43.5*
	>5 years	15.3	2.9	16.1	17.9	27.5	7.0	**3.9**	**9.3**	29.3	**34.8**
Deployment in the last 12 months	No	14.2	4.1*	15.0	17.9	28.9	7.1*	3.9*	12.1	30.5	34.6
	Yes	15.3	**2.3**	15.3	16.7	40.1	**17.2**	**12.0**	16.2	42.1	51.1
Military branch	Army	13.2	**2.8** **	13.5	15.6*	37.4***	6.6	4.1	12.9	37.4**	43.0
	Air force	14.9	**3.9**	15.9	**18.4**	**26.1**	10.0	7.0	11.5	**27.5**	32.4
	Navy	18.4	**5.3**	19.3	**22.7**	**24.5**	8.5	1.6	14.9	**30.9**	33.0

^a^Verbal SH includes repeated sexual comments or repeated verbal unwanted verbal sexual attention,

^b^ MST includes all forms of sexual stressors

^*^ *p* < 0.05,

^**^ *p* < 0.01,

^***^ *p* < 0.001,

^#^ non computable

^c^ repeated unwanted verbal attention and sexual coercion is too rare to conduct bivariate analysis for men.

Results also indicate a significant effect of the work environment. Men working in the Navy were more likely to report repeated SA while women in the Army were more likely to report repeat sexism contributing to an increased verbal SH. In addition, women who had been deployed in the last 12 months were more likely to report repeated unwanted verbal attention and sexual coercion.

Because of differential associations between sexual stressors and individual and work environement factors, many factors that were related to specific sexual stressors were not longer associated with MST. For men, only sexual orientation and military branch were associated with MST, while for women, less time serving in the military (less than 5 years), lower level of education and military rank were related to MST.

Multivariate analyses indicated greater likelihood of experiencing verbal SH among servicemen who have sex with men and among women officers. The odds of SA were elevated among younger men and reduced among more educated women. Finally, the odds of MST were increased among men who had same sex partners and female officers but lower among more educated women.

For men and women alike, the work environment was significantly related to their probability of experiencing verbal SH, SA as well as MST. Women in the Air Force were less likely than women in the Army to experience verbal SH while men in the Navy were more likely to have experienced SA (this variable was not included in the final model due to model instability). The odds of verbal SH were 80% and 20% higher among men who worked in units where sexual comments and sexual oppression (unwanted attention, coercion and assault) were more prevalent (above the median), and were 50% and 40% higher when men worked in units with low acceptance of increased feminization of the army and higher female representation. Likewise, the odds of SA were elevated among men working in units with higher female representation. Altogether, the odds of men experiencing MST were elevated in units with higher prevalence of sexual comments, and lower acceptance of female representation. For women, the odds of SA were particularly high (OR = 5.7) among women working in units with a higher prevalence of sexual oppression, while at the same time these women had reduced odds of verbal SH. Likewise, the odds of SA were lower when women worked in units with higher prevalence of sexism and low acceptance of military feminization. Altogether, the odds of experiencing any sexual stressor (MST) were lower among women working in units with low female representation and low acceptance of female representation (Table 4A and 4B).

**Table 4:** **a:** Multivariate analysis of *Verbal SH*, *Sexual Assault*, *and MST* in the military work environment in the last 12 months among men. **b:** Multivariate analysis of *Verbal SH*, *Sexual Assault Sexual Oppression and MST* in the military work environment in the last 12 months among women.

		Verbal SH ^a^		Sexual Assault^b^		MST^c^	
		OR	95%CI	OR	95%CI	OR	95%CI
Age	<25	ref.		ref.		ref.	
	25–29	1.3	[0.6,2.9]	0.8	[0.4,1.5]	1.3	[0.6,2.6]
	> = 30	1.6	[0.7,3.6]	**0.3** ^*^	[0.1,0.8]	1.2	[0.6,2.4]
Cohabitation with current partner:	Everyday	ref.		ref.		ref.	
	Not every day	1.4	[0.8,2.6]	1.6	[0.6,4.5]	1.4	[0.8,2.4]
	No current partner	1.0	[0.6,1.7]	2.4	[0.7,7.7]	1.2	[0.9,1.7]
Level of education	Less than High school	ref.		ref.		ref.	
	High school graduation	1.5	[0.9,2.3]	0.7	[0.3,2.0]	1.4	[0.8,2.5]
	>High school	1.7	[0.9,3.5]	0.3	[0.1,1.8]	1.5	[0.7,3.4]
Financial situation:	No problem	ref.		ref.		ref.	
	Difficult/Just enough	1.2	[0.5,2.5]	1.4	[0.4,4.7]	1.1	[0.5,2.7]
Sexual orientation:	Heterosexual	ref.		ref.		ref.	
	Bi/Homosexual	**3.5** ^**^	[1.4,8.7]	2.7	[0.5,13.4]	2.8*	[1.1,7.5]
	Never had sex	0.3	[0.0,4.9]	1.0	[1.0,1.0]	0.3	[0.0,3.0]
Army rank	Enlisted personnel	ref.		ref.		ref.	
	Non commissioned officer	0.9	[0.5,1.8]	0.9	[0.4,2.3]	0.8	[0.5,1.4]
	Officer	1.3	[0.6,2.8]	2.6	[0.9,7.4]	1.4	[0.7,2.7]
Place of birth	Mainland France	ref.		ref.		ref.	
	Overseas France/Foreign	1.4	[0.7,2.8]	1.4	[0.8,2.5]	1.4	[0.7,2.8]
Deployment in the last 12 months	No	ref.		ref.		ref.	
	Yes	1.3	[0.9,1.8]	0.7	[0.4,1.3]	1.1	[0.9,1.4]
Higher % sexual comments		**1.8** ^***^	[1.5,2.3]	0.9	[0.7,1.3]	1.8***	[1.5,2.1]
Higher % sexual oppression^d^		**1.2** ^***^	[1.1,1.3]	1.7***	[1.3,2.2]	1.3***	[1.1,1.5]
Low acceptance of increasing female representation in the army		**1.5** ^**^	[1.2,2.0]	1.2	[0.7,2.0]	1.4*	[1.0,1.9]
High unequal sexual norms		1.2	[0.9,1.4]	0.9	[0.7,1.2]	1.2	[0.9,1.4]
Low social cohesion		1.0	[0.8,1.2]	1.1	[0.8,1.5]	1.0	[0.8,1.2]
Higher Female representation		**1.4** ^*^	[1.1,1.9]	**1.7** ^***^	[1.2,2.4]	1.3	[1.0,1.8]
N		1258		1243		1256	
Age	<25	ref.		ref.		ref	
	25–29	0.6	[0.3,1.5]	1.7	[0.9,3.2]	0.7	[0.3,1.9]
	> = 30	0.7	[0.3,2.3]	0.7	[0.2,1.9]	0.8	[0.3,2.1]
Cohabitation with current partner:	Everyday	ref.		ref.		ref.	
	Not every day	2.2	[0.6,8.0]	1.7	[0.4,7.3]	1.5	[0.5,4.6]
	No current partner	1.8	[0.8,4.2]	2.0	[0.7,5.8]	1.4	[0.7,2.7]
Level of education	< High school	ref.		ref.		ref.	
	High school graduation	0.7	[0.2,2.1]	**0.3** ^*^	[0.1,1.0]	0.6	[0.2,1.4]
	>High school	0.3	[0.1,1.3]	0.1***	[0.0,0.4]	0.2**	[0.0,0.6]
Financial situation	No problem	ref.		ref.		ref.	
	Difficult/Just enough	0.7	[0.3,1.6]	1.1	[0.4,3.3]	0.7	[0.4,1.4]
Sexual orientation	heterosexual	ref.		ref.		ref.	
	Bi/Homosexual	1.4	[0.3,5.6]	0.8	[0.2,3.5]	1.4	[0.4,5.2]
	Enlisted personnel	ref.		ref.		ref.	
Army rank	Non commissioned officer	1.0	[0.4,2.8]	0.4	[0.1,1.6]	0.9	[0.3,3.0]
	Officer	**4.6** ^**^	[1.6,13.7]	1.0	[1.0,1.0]	4.3**	[1.6,11.2]
Place of birth:	Mainland France	ref.		ref.		ref.	
	Overseas /Foreign	0.7	[0.2,2.9]	1.5	[0.2,12.7]	0.7	[0.2,2.4]
Mission in the last 12 months	No	ref.		ref.		ref.	
	Yes	1.3	[0.5,3.2]	0.9	[0.1,7.0]	1.6	[0.4,6.6]
Higher % sexual comments		0.8	[0.4,1.7]	**0.5** ^*^	[0.3,0.9]	0.6	[0.3,1.2]
Higher % sexual oppression^d^		**0.6** ^**^	[0.4,0.9]	**5.7** ^***^	[3.6,9.1]	1.0	[0.8,1.4]
Low acceptance of increasing female representation in the army		**0.6** ^*^	[0.4,0.9]	1.0	[0.6,1.7]	0.5*	[0.3,0.8]
High unequal sexual norms		1.1	[0.5,2.2]	0.5*	[0.2,0.9]	0.9	[0.5,1.8]
Low social cohesion		0.9	[0.6,1.4]	1.0	[0.6,1.6]	1.1	[0.7,1.8]
Higher Female representation		0.6	[0.3,1.3]	0.5	[0.2,1.0]	**0.5** ^*^	[0.2,1.0]
N		230		216		229	

Table 4a: ^a^ Verbal SH = repeated sexual comments or repeated unwanted verbal sexual attention ^b^ sexual assault = touching, attempted or forced sex including with object or finger ^c^. MST includes all sexual stressors ^d^ sexual oppression = repeated unwanted verbal sexual attention, sexual coercion or sexual assault

Exponentiated coefficients; 95% confidence intervals in brackets; ^*^ *p* < 0.05, ^**^ *p* < 0.01, ^***^ *p* < 0.001

Table 4b: ^a^ Verbal SH includes repeated sexual comments or repeated unwanted verbal sexual attention ^b^ sexual assault includes touching, attempted or forced sex including with object or finger ^c^ MST includes all sexual stressors ^d^ sexual oppression = repeated unwanted verbal sexual attention, sexual coercion or sexual assault

Exponentiated coefficients; 95% confidence intervals in brackets; ^*^ *p* < 0.05, ^**^ *p* < 0.01, ^***^ *p* < 0.00.

## Discussion

In line with previous studies conducted in the US military [23, 24], we found that MST is a common experience for women in the French military. While patterns of sexual stressors were comparable between men and women, the gender gap was profound, with women experiencing multiple forms as well as the most severe forms of sexual stressors. Such results display the realities of MST in France, although the nature, meaning and consequences of these experiences are likely different for men and women [6, 12, 34].

While published population-based estimates are unavailable in France, our 12 months prevalence of SA (12.6% among women and 3.5% among men) appears to be higher than in the French civilian population, based on the 2016 Virage study reporting 12 month prevalence rates ranging from 2.9% to 5.5% among women 20–49 years and from 1.16% to 1.5% among men of the same age groups (Virage) [29]. Comparisons with other military populations suggest higher MST incidence in the French military (men and women alike), but similar patterns of sexual stressors [26–34]. For both sexes, our MST prevalence rates were close to 10 percentage points higher than US estimates, and far exceeded the 5.7% prevalence reported among Korean military women [29]. Conversely, our 12 months MST prevalence for women (36.7%) was lower than the 84% reported among women cadets and officers in the Swedish army, although this estimate was based on a 24-month time frame [28]. SA was also more prevalent in our study compared to the 2016–2018 US estimates for servicemen (0.6%-0.7%) but closely aligned with the experiences of US servicewomen (4.3%- 6.2%) [26]. Less than 2% of women in our study reported attempted or forced penetrative sex, versus 3% among US military women and 1.9% among Swedish army servicewomen (albeit over 24 months).

Differences in MST estimates across studies, likely reflect variations in definitions and measures [2, 8, 24], survey procedures [2, 8] and social context [28]. The COSEMIL MST metric explored several of the SEQ DoD dimensions but included fewer and modified items to adapt to the cultural context as suggested by Wasti and colleagues [35] and to allow comparisons with the national Virage estimates [32]. In particular, our measure of unwanted sexual attention only included verbal encounters, as we categorized all physical forms of unwanted sexual contact in a seperate category of SA, to explore the continuum of harm. In addition, unlike the revised SEQ DoD [36], the COSEMIL study did not evaluate the perceived severity of hostile environment events, which may have contributed to an overestimation of MST, although we only counted repeated events as MST. Comparability of study results may also be compromised by sample selection. Unlike most prior research which suffers high non-response rates [24], the COSEMIL study used a probability sampling design across military branches and ensured high response rate to reduce selection bias [30]. Beyond measurement and study procedures, differences in MST estimates across studies may reflect differences in social context and workplace environment [4]. Substantial variation in SH is observed across the 28 EU member States, as evidenced by Latcheva, showing higher SH prevalence in France and Northern Europe than other regions [37]. Such cross-cultural comparisons are interesting to consider within the military context, which shares a number of common features, including organizational structure and masculinist culture [19, 24] but are limited in scope given the paucity of research conducted outside of the United States. Using the SEQ DoD construct, a Swedish study substantiates the relevance of the SEQ DoD’s three dimensional SH construct in a European context but assesses MST over a 2 year period, limiting the potential for comparison [28].

Beyond overall prevalence rates, this study provides an opportunity to explore SH as a continuum of harm [24, 25] distinguishing experiences of verbal SH from SA. While we show significant overlap between these events, we also find differences in their predictors leading to null findings when examining the aggregated measure of MST as associations related to verbal SH and SA cancelled each other out.

The opportunity to explore MST in th French military context adds to existing research mostly conducted in the US, providing an opportunity to examine the extent to which predictors of SH are similar or different across military contexts. Consistent with a number of studies [4], we found that the workplace environment was the strongest predictor of verbal SH and SA in our military population [27], although these effects differed by gender and by sexual stressor. As previously described [38], deployment seemed to increase the risk of SA among women, although the association was no longer significant after multivariate adjustments. Supporting the Person Situation model, job gender context and group level exposures had different effects for men and women [4], and we also found these effects to differ by sexual stressor. While sexist and violent environments were related to individual experience of sexual stressors for men, low acceptance of female representation was associated with reduced MST among women, which was unexpected. These differential effects by sexual stressors and gender have not been reported in previous literature, although the small female sample size limits the interpretation of these findings. Willness’ systematic review indicates smaller job gender context effects in the military than in other workplace environments, which could be explained by differential effects by gender, or by different definitions of SH, combining different sexual stressors [4]. Other conditions of the workplace including social cohesion or unequal gender norms were not statistically associated with individual experiences of sexual stressors in our study.

Beyond situational factors, we also identified a number of individual and interpersonal characteristics associated with verbal SH and/or SA. Specifically, less educated women were more likely to report instances of SA, as was also the case for men born outside of mainland France (although non significant in multivariate analysis). These results corroborate Bell’s observation of sociocultural power conditions heightening the risk of sexual victimization in military populations [27]. On the contrary, women officers were more likely to report instances of sexual coercion and repeated unwanted verbal sexual attention. The greater exposure to SH among women who do not conform to the gender order has been noted in prior studies in the general population, including the FRA study among women across 28 EU states which indicates that women in the highest occupational groups and with higher levels of education are more likely to experience SH than others [37]. In line with the sanctioning of gender non-conformity, we also found that men who had sex with men were more likely to report verbal SH, which potentially echoes the nature of sexual comments, shown to be commonly homophobic in a US military study [39]. The increased risk of overall MST among men with male sexual partners is consistent with prior studies reporting increased risk of MST among LGBT individuals in the US military [40–42].

In addition to the SH measurement concerns discussed above, this study has a number of limitations, including the small female sample size and small sample size of LGBT individuals, resulting in large confidence intervals. Lack of information about acceptance of and retaliation against SH in the workplace, shown to be strongly predictive of MST [16, 23] also limits the interpretation of the environmental context condusive of MST. In addition, the cross-sectional nature of the study limits the understanding of the continuum of harm and prevents any causal interpretation although our conclusions are mostly consistent with findings of prospective studies.

Despite these limitations, the use of measures of MST along the continuum of harm, the use of a probability sample of the military population, and the systematic gender exploration of predictors of different dimensions of MST all constitute important strengths of this analysis. Given the public health implications of SH, especially in military populations, where servicewomen have been shown to suffer greater mental health sequealea following SH than in civilian populations, we suggest that the high prevalence of SH in the French military calls for programmatic action to prevent MST as well as screening programs and services for those who have experienced MST in the French military. The implementation of such programs have been effective in the US military [43], but have not been evaluated in the European context, including France, where a ministry of defense program (Themis program) was created in 2014 and revised in 2018. Such efforts not only address a human’s rights imperative but also have positive public health implications in reducing the health sequelae related to these events.
